# Rheological properties of milk-based desserts with the addition of oat gum and κ-carrageenan

**DOI:** 10.1007/s13197-019-03983-4

**Published:** 2019-08-01

**Authors:** Piotr Zarzycki, Aleksandra Elżbieta Ciołkowska, Ewa Jabłońska-Ryś, Waldemar Gustaw

**Affiliations:** Department of Plant Food Technology and Gastronomy, University of Life Sciences in Lublin, ul. Skromna 8, 20-704 Lublin, Poland

**Keywords:** Dairy dessert, Flow behaviour, Mechanical spectra, Texture

## Abstract

A growing interest in development of milk desserts with good nutritional and rheological properties can be observed. A good and stability rheological as well as nutritional properties of such desserts can be provided by applying suitably composed gum mixtures. In this work, the effect of 0.1% κ-carrageenan addition on the rheological properties of based-milk desserts with different oat gum concentrations (0.1, 0.3 and 0.5%) was investigated. All milk desserts tested in presented study showed a time dependent and shear-thinning flow behavior. The mechanical spectra were characterized by storage module (G’) greater than loss module (G”), typical for viscoelastic materials such as gels and dispersions. The incorporation of 0.1% κ-carrageenan into milk dessert with different oat gum concentrations allows to obtain stronger gel structure compared to milk dessert with separate oat gum addition. It can be also observed that desserts systems with the 0.1% κ-carrageenan had more stable viscoelastic properties. Moreover, the use the κ-carrageenan addition caused an increase in consistency coefficient (K) and decreased in n-value for Ostwald de Waele rheological model. Combined addition of oat gum and carrageenan allows to obtain milk dessert with stronger texture. The hardness of milk desserts range from 0.32 to 0.49 N for desserts without κ-carrageenan addition and from 0.513 to 0.557 N for desserts with κ-carrageenan. The high synergistic effect of composed gum mixtures on rheological properties of milk dessert occurs at 0.1% oat gum and 0.1% κ-carrageenan concentration.

## Introduction

Milk-based desserts due to their sensory and nutritional characteristics are widely consumed by different groups of consumers, such as children, adults and elderly people (Nastaj et al. 2007; Toker et al. 2013; Szwajgier and Gustaw 2015; Aguilar-Raymundo and Vélez-Ruiz 2018). Basically, milk-based desserts are formulated with whole and/or skimmed milk, different kind of thickeners such as starch and hydrocolloids, sucrose, colorants and aroma. The rheological and textural properties of these products are very important in terms of industrial manufacturing, preparation in the kitchen, consumer acceptance, and nutritive characteristics (Reis et al. 2011; Toker et al. 2013). In general, this type of product shows time dependent and shear-thinning flow behavior, and viscoelastic properties typical for weak gels. However, noticeable differences in rheological properties can be found in model systems with different compositions (Tárrega and Costell 2006; Szwajgier and Gustaw 2015; Aguilar-Raymundo and Vélez-Ruiz 2018). When new components or its combinations are incorporated into products, the effect of such modification on rheological and textural properties should be researched.

One of the typical hydrocolloid used as a thickener in dairy/milk desserts is carrageenan. Carrageenan consists of a linear sulphated polysaccharide, namely O-β-D-galactopyranosyl-4-sulfate-(1-4)-O-3,6-anhydro-α-D-galactopyranosyl-(1-3), obtained from red seaweeds. The most commonly used carrageenan fractions include κ-, ι- and λ-carrageenan (Gustaw 2008a). κ-carrageenan forms strong and brittle gels, ι-carrageenan gives delicate and elastic gels, whereas λ-carrageenan forms liquid and viscous solutions (Gustaw and Mleko 1998). Several studies have been focused on analyzing κ-carrageenan to improve the rheological properties of dairy desserts. According to Reis et al. (2011) κ-carrageenan due to its complex with κ-casein micelles of milk can generating a thickening effect up to 10 times higher than its effect in water. Toker et al. (2013) evaluated the effect of xanthan, guar gums, carrageenan, alginate, as well as their mixtures, on rheological properties of a dairy dessert, and found that κ-carrageenan is one of the most effective hydrocolloid influencing on rheological parameters of the dairy dessert.

In recent years, nutritional trend for foods enrichment has become very important. One of the methods of improving nutritional properties is dietary fibre supplementation, which is used practically in all food product categories. Dietary fiber, such as (1 → 3), (1 → 4)-D-β-glucan (β-glucans), is well known to confer significant health-promoting properties. β-glucans, mostly found in oat and barley, exhibit many health benefits including the reduction of colon cancer risk, reduction of total and low-density lipoprotein content and the regulation of post-prandial blood glucose and insulin levels (Sayar et al. 2006; Weickert et al. 2006; Keith et al. 2014; Patel 2015). The ability of β-glucans to impart gut viscosity is one of the main hypothesis, apart from colonic fermentation, explaining the health-promoting effect of β-glucans (Anttila et al. 2004; Queenan et al. 2007). The increased gut viscosity is believed to be one of the key mechanism responsible for lower absorption of sugars and bile acid. The role of viscosity of β-glucans in reducing the postprandial glucose and insulin rise, as well as lowering of plasma cholesterol is well documented in literature (Wood et al. 1994; Reppas et al. 2009; Wolever et al. 2010). Apart from its nutritional benefits, β-glucans has been often proposed to use in food formulation due to their rheological characteristics; i.e., gelling properties and viscosity enhancement (Gustaw 2008b; Gustaw and Szwajgier 2012; Lazaridou et al. 2014).

The use of two or more gums in the product formulation is wildly widespread in the food industry due to the synergistic effect of gum mixture. The proper gum combination may improve product quality including nutritional properties, and also provide economic benefits (Toker et al. 2013; Qasem et al. 2017). Previous studies on mixtures of milk proteins with oat gum, showed a segregative phase separation, which resulted in a significant deterioration of rheological properties of products containing higher concentrations of oat gum (Gustaw 2008b; Gustaw and Szwajgier 2012). To best of our knowledge, there are many studies about the specific and synergic effect of gums in different food product categories, also in dairy desserts (Reis et al. 2011; Toker et al. 2013; Lazaridou et al. 2014), but there is a lack of information in literature about the effects of combination use oat gum and κ-carrageenan in milk desserts on rheological and textural product properties.

Thus, based on the aforementioned studies, the aim of this study was to investigate the effect of oat gum concentrations on the rheological properties of milk-based dessert model systems containing κ-carrageenan.

## Materials and methods

### Materials

The following materials were used in the study oat gum obtained from high fibre oat bran with (1 → 3), (1 → 4)-D-β-glucans contents of 7.50 ± 0.6% (PZZ Kruszwica, Poland), skim milk powder (SMP) containing 34% of protein (SM Gostyń, Poland), κ-carrageenan (Sigma, USA), modified tapioca starch T-440 (E-1422, Avebe, Veendam, Holand) and sucrose.

### Extraction of oat gum from oat bran (GO)

The oat gum was isolated from the oat bran using method of Beer et al. (1996). 6 g of oat bran was blended with 90 ml of distilled water at 40 ^°^C, and mixed for 15 min (magnetic stirrer type MS 11 HS, Wigo, Poland, 500 rpm). Then, the pH of mixture was adjusted to 10 with 20% sodium carbonate and stirred for addition 30 min (40 ^°^C, 500 rpm). The mixture was centrifuged (Sigma 4K15, Polygen, Poland, 2000 × *g*, 10 min), and the supernatant was decanted (E1). The residue was reextracted at the condition described above and extract (E2) was obtained. The mixture of two extracts (E1 and E2) were cooled to 20 °C, pH was adjusted to 4.5 with 20% (v/v) hydrochloric acid and then centrifuged (2000 × *g*, 10 min). The supernatant gum extract was cooled to 10 ^°^C, mixed with absolute ethanol (1:1 v/v) and centrifuged (2000 × *g*, 10 min). The gum solids were collected, dried (freeze dryer type Alpha 1-2 LD plus, Christ, Germany) and ground (GM 200, Retsch GmbH, Germany) to pass a 250 µm screen. The obtained oat gum isolate contained 82.0 ± 0.3% of β-glucan. The content of (1 → 3), (1 → 4)-D-β-glucan was determined with the McCleary method (AACC Method 32–23), using Megazyme enzymes and methodological procedures (Megazyme International, Wicklow, Ireland).

### Composition and preparation of the milk dessert

The base formulation for the milk dessert consisted of 11.75% of skim milk powder, 3% of modified tapioca starch and 8% of sucrose. This formula was enriched by addition of oat gum (0, 0.1, 0.3, 0.5%) and κ-carrageenan (0 or 0.1%). Table 1 shows the compositions and identification codes for all the formulations. The following procedure was used for the milk dessert preparation. First, all ingredients (skim milk powder, tapioca starch, sucrose oat gum and κ-carrageenan) were weighted, according to formula, and dispersed slowly in the correspondent 0.1 mol/L NaCl solution to obtain 30 mL, for 30 min under constant stirring at 70 ^°^C (magnetic stirrer MS 11 HS, Wigo, Poland). Subsequently, the mixture was heated up to 95 ^°^C, kept for 15 min on a heating laboratory bath (W-215, Laboplay, Poland) and then each formulation was allowed at cool down until room temperature. After that, the milk desserts were transferred into beakers and storage (24 h, 4 °C) prior to texture measurements.

**Table 1:** Composition and identification codes for milk dessert formulation

Code	Composition of milk dessert (%)					
	SMP	Modified tapioca starch	κ-carrageenan	Saccharose	Oat gum	0.1 mol/L NaCl-solution
1-Con	11.75	3	0	8	0	77.25
2-OG0K	11.75	3	0.1	8	0	77.15
3-OG01	11.75	3	0	8	0.1	77.15
4-OG01K	11.75	3	0.1	8	0.1	77.05
5-OG03	11.75	3	0	8	0.3	76.95
6-OG03K	11.75	3	0.1	8	0.3	76.85
7-OG05	11.75	3	0	8	0.5	76.75
8-OG05K	11.75	3	0.1	8	0.5	76.65

SMP—Skim milk powder, samples description; Con—control (0%-oat gum, 0%-κ-carrageenan); OG0(K), OG01(K), OG03 (K), OG05(K)—samples with different oat gum concentration, respectively 0, 0.1, 0.3 and 0.5%, K-0.1% κ-carrageenan addition

### Viscoelastic behavior of milk dessert

Rheological evaluations were completed using a dynamic rheometer RS 300 (ThermoHaake, Karlsruhe, Germany) monitored by Haake RheoWin 3.61, using a concentric cylinder geometry (Z41). The frequency sweep was conducted at samples temperature 20 ^°^C, regulated by DC 30 circulator (ThermoHaake, Karlsruhe, Germany), with oscillation frequencies ranging from 0.1 to 100 Hz, in which the values of the storage modulus (G’) and the loss modulus (G”) were recorded as a function of frequency. Three replicates were completed for each sample.

### Flow behavior of milk dessert

The flow curves of milk desserts were measured by recording shear stress values when shearing the samples with a linear increasing shear rate from 1 to 200 s^−1^ for a period of 60 s. Data were fitted to the Ostwald de Waele (Eq. 1) and Herschel–Bulkley (Eq. 2) models, where σ (Pa) is shear stress, K (Pa s^n^) is consistency coefficient, γ (s^−1^) is the shear rate, σ_o_ (Pa) is yield stress, and n which is flow index is dimensionless, using Haake RheoWin software, version 3.61 (ThermoHaake, Germany).

1 $$ \upsigma = {\rm K} {\upgamma}^{\rm n} $$

2 $$ \upsigma = \upsigma_{o} + {\rm K} \upgamma^{\rm n} $$

Time depending behavior of milk dessert was evaluated by recording up and down shear stress values between shear rates of 1–200 s^−1^ for a period of 60 s, and in a reverse sequence for the same time. The thixotropic area (A_T_) showing the difference between areas under the upstream data point curve and under the downstream data point curve were obtained using Haake RheoWin software, version 3.61.

### Texture analysis of milk dessert

The texture profile analyses test (TPA) were performed, in six repeats, by a penetration test using stainless steel cylindrical probe (15 mm diameter) at the crosshead speed of 1 mm/s and 70% deformation. The TA-XT2i Texture Analyser (Stable Microsystems, Goaldming, UK) with software Texture Export ver. 1.22 was used in the study. Five parameters were measured during TPA: hardness (N), springiness (m), cohesiveness (no unit), gumminess (N) and chewiness (J).

### Statistical analysis

Eight milk desserts systems were prepared, following a factorial design: 2 × 4; with two concentration levels of κ-carrageenan (0 and 0.1%) and four concentration levels of oat gum (0, 0.1, 0.3, and 0.5%) The levels of the factors were chosen according to the preliminary research on milk desserts. Three replicates were completed for each system (individual experimental units). The analyses were carried out 24 h after milk desserts preparation. Statistical analysis was performed using STATISTICA 13.0 (Stat-Soft, Cracow, Poland). Duncan test (*p* ≤ 0.05) was used to identify statistically significance of differences between mean values.

## Results and discussion

### Viscoelastic behavior response

**Fig. 1:**
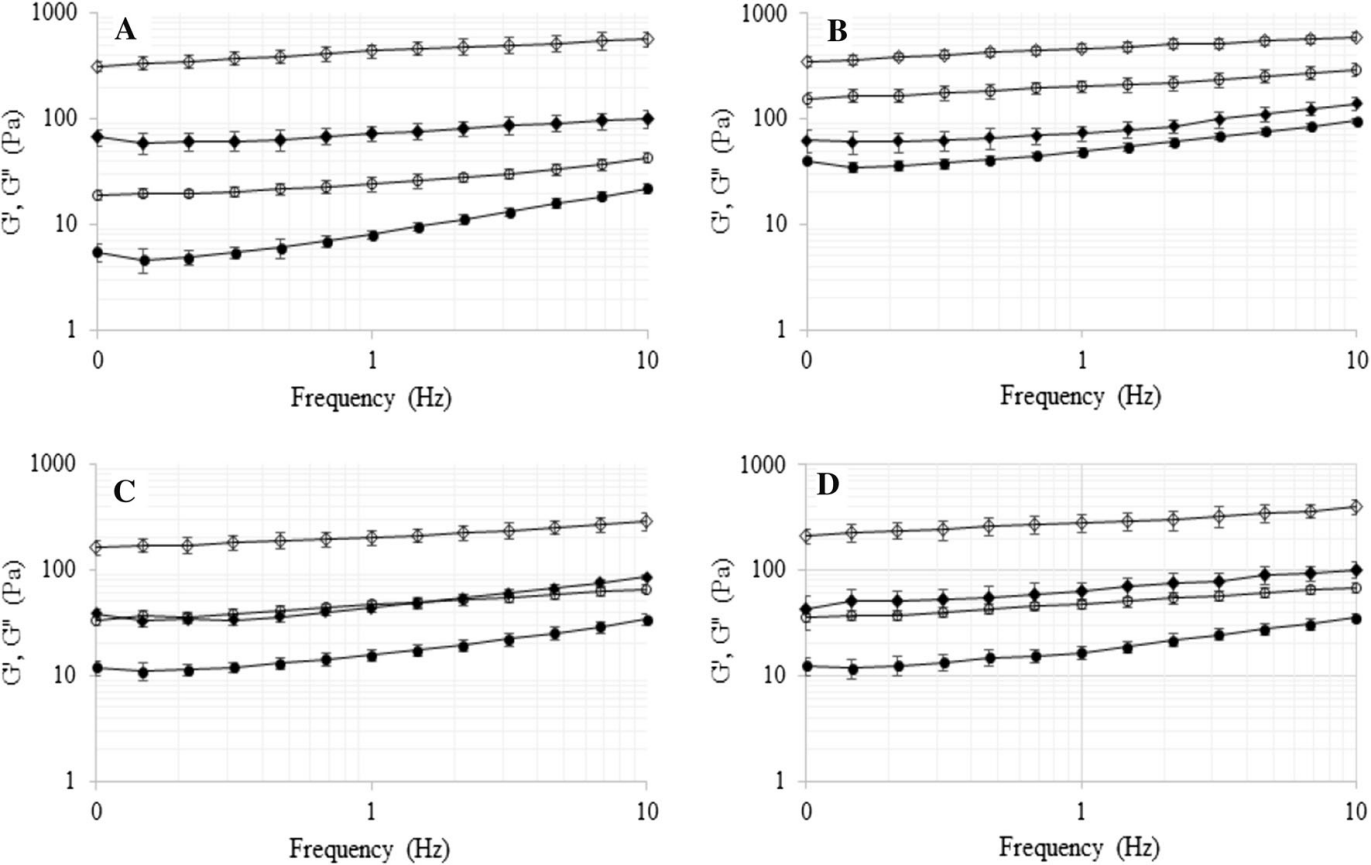
Mechanical spectra for milk dessert with 0% (“open circle”, “filled circle”) and 0.1% (“open diamond”, “filled diamond”) addition of κ-carrageenan: **a** milk dessert without oat gum, **b** milk dessert with 0.1% oat gum concentration, **c** milk dessert with 0.3% oat gum concentration, **d** milk dessert with 0.5% oat gum concentration. Empty symbols for the storage modulus (G’) and filled symbols for the loss modulus (G”)

Mechanical spectra obtained for milk desserts with different concentrations of oat gum and κ-carrageenan are shown in Fig. 1a–d, with both storage (G’) and loss modulus (G”). The mechanical spectra, for all samples, were characterized by G’ values greater than G” values, that behavior is typical for viscoelastic materials such as gels and dispersions (Szwajgier and Gustaw 2015; Aguilar-Raymundo and Vélez-Ruiz 2018). The weak dependence on frequency of both modulus (G’ and G”), characteristic for weak-gel systems, was also observed. Moreover, the addition of κ-carrageenan to the milk desserts decreased the frequency dependence of loss modulus (G”). The slight increase of both modulus observed in this study, can be attributed to the absence of binding agents (gluten, starch) in the dairy dessert samples (Sivaramakrishnan et al. 2004). In none of the tested samples a decrease in the G’ value with increasing frequency was observed, which could indicating the destruction of the gel matrix.

As expected, the G’ and G” values were higher for milk-dessert with oat gum addition compared to control (Fig. 1a–d), however, there was no linear relationship between the concentration of oat gum and both modulus value (G’ and G”). Among the desserts without κ-carrageenan addition, the highest G’ and G” values were observed for 0.1% oat gum concentration (Fig. 1b). The G’ and G” values for dessert with 0.3 and 0.5% oat gum concentration (Fig. 1c and d, respectively) were much lower compared to values noted for 0.1% oat gum concentration, but still higher compared to control (Fig. 1a–d).

This behavior can be explained by the occurrence of segregative phase separation between probably between casein and oat gum (Gustaw 2008b; Gustaw and Szwajgier 2012). Aqueous biopolymer mixtures are in most cases characterized by a thermodynamic incompatibility that leads to a macroscopic phase separation (Mekhloufi et al. 2005). According to Bergfeldt et al. (1996) the phase separation of two biopolymers in a common solvent may be divided into two main categories:  associative and segregative phase separation. In an associative phase separation, both biopolymers are enriched in one of the separating phases with the other phase containing mostly solvent. This type of phase separation is often obtained for oppositely charged biopolymer mixtures. In a segregative phase separation, two polymers are separated into two different phases. This is the case mainly for two nonionic biopolymers, two similarly charged polyelectrolytes, or a polyelectrolyte plus a nonionic biopolymer.

It may be observed that the addition of 0.1% κ-carrageenan results in higher strengthening of the gel structure (higher G’ and G” values observed), compared to 0.1% addition of oat gum (Fig. 1a and b). These data are in agreement with previous studies presented by Toker et al. (2013) in which dairy desserts containing carrageenan had the highest G’, G”, and lowest tan δ (G”/G’) values among dessert with different kind of gum addition (carrageenan, alginate, guar and xanthan gums and their combinations). Moreover as showed in presented study, the addition of 0.1% κ-carrageenan to milk dessert with different oat gum concentrations allows to obtain stronger gel structure compared to milk dessert with separate oat gum addition (higher G’ and G” values observed). However, similar pattern to dessert samples with separate oat gum concentration was observed, that means the highest G’ and G” values were obtained for milk dessert with 0.1% oat gum concentration (Fig. 1b). An increase in oat gum concentration to 0.3 and 0.5% (Fig. 1c and d, respectively) resulted in decrease G’ and G” values, but the noted values for both moduli were higher compared to dessert with separate oat gum addition, regardless of the amount of oat gum concentration. Probably carrageenan reduced the negative effect of the separation phase between milk proteins and oat gum on the rheological properties of the tested dairy desserts, but 0.1% carrageenan concentration did not completely eliminate the effect of this phenomenon on the deterioration of rheological properties analyzed samples.

The results obtained in this study indicate that, the addition of oat gum has a greater impact on viscous than elastic properties of milk-based desserts compared to κ-carrageenan addition. Increasing the concentration of oat gum above 0.1% leads to a weakening of the gel structure. The use of κ-carrageenan gives, the possibility of using higher concentration of oat gum in milk dessert, up to 0.5%.

### Flow behavior response

**Fig. 2:**
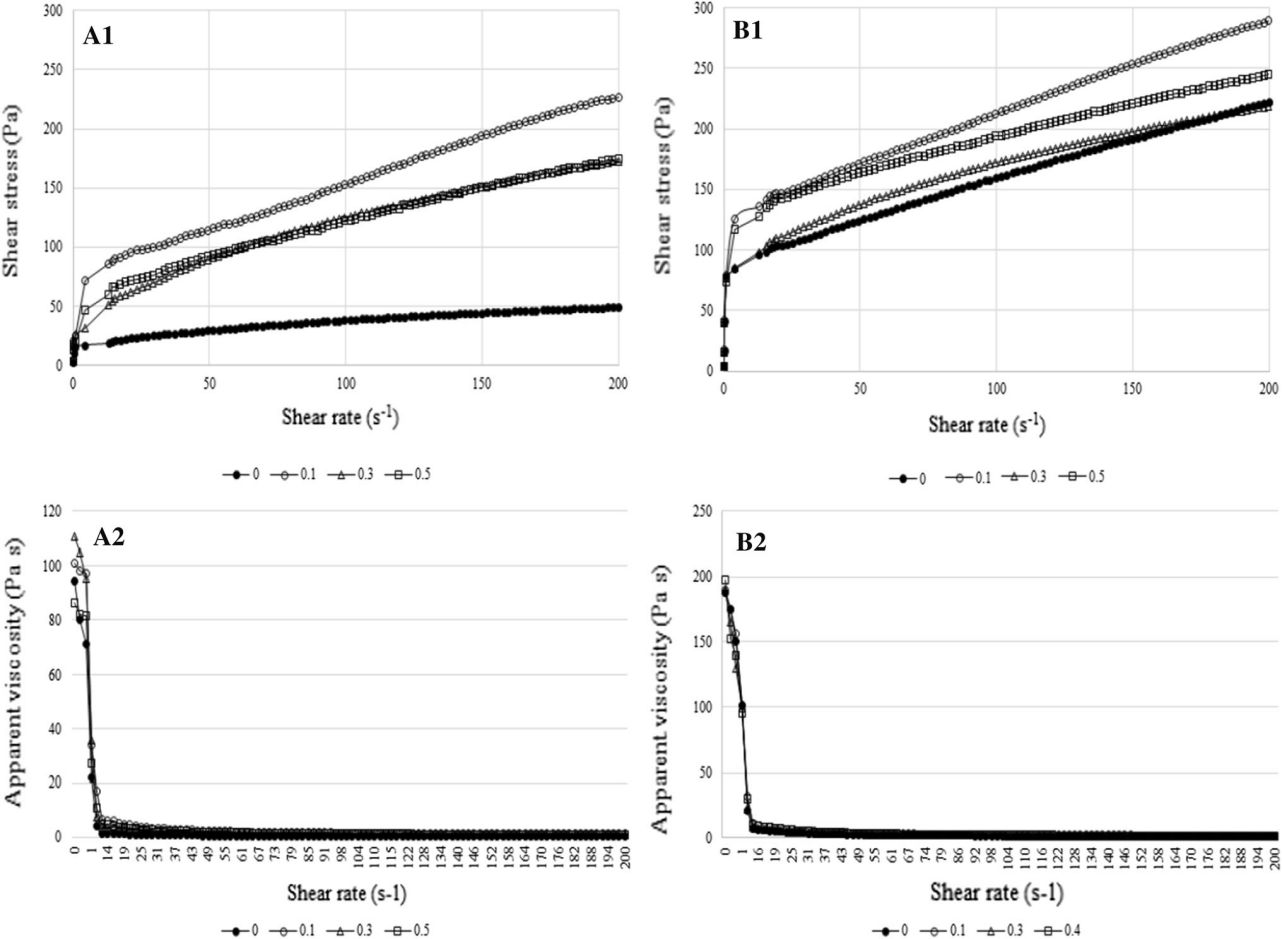
Flows curves (A1, B1) and viscosity curves (A2, B2) of milk desserts with different content of oat gum (0, 0.1, 0.3, and 0.5): **a** and **b** correspond to milk dessert with 0 and 0.1% addition of κ-carrageenan respectively

All milk desserts, obtained in this study, exhibited time dependent (thixotropic or antithixotropic) and shear-thinning flow behavior. Flow curves and viscosity curves for milk dessert are shown in Fig. 2, correspond to milk dessert with 0% (A) and 0.1% (B) addition of κ-carrageenan respectively. The flow curves showed a similar pattern although clear differences were observed among them, depending on oat gum concentration and κ-carrageenan additions. The flow curves of eight analyzed milk dessert showed a non-Newtonian response, exhibiting a yield stress and a shear thinning flow–increase in shear stress (decrease in apparent viscosity) with increase in shear rate. That behavior is usually observed in weak gel systems, witch in accordance with previously described results, shown in Fig. 1a–d. Similar flow behavior have been reported by other researchers for milk-based desserts with addition of κ-carrageenan, starch, brewery malts and chickpea flour (Mleko and Gustaw 2002; Szwajgier and Gustaw 2015; Aguilar-Raymundo and Vélez-Ruiz 2018).

The addition of oat gum allows to obtained milk dessert with higher value of shear stress compared to control (samples 3, 5, 7 vs 1), but it should be noticed that the highest shear stress values were observed for milk dessert with 0.1% oat gum concentration. With increase oat gum concentration above 0.1% the shear stress decreases. That pattern occurs for both tested type of milk desserts–with or without κ-carrageenan addition (Fig. 2a and b, respectively). Additionally, the highest shear stress values for milk desserts prepared with the addition of κ-carrageenan were observed (Fig. 1a and b), when the samples with the corresponding oat gum concentration are compared. The shear stress value for milk desserts without κ-carrageenan, at the highest shear rate applied 200 s^−1^, were 226.2 Pa, 174.9 Pa, 172.6 Pa and 49.4 Pa, respectively for 0.1%, 0.3%, 0.5% oat gum concentration and sample control (sample 3,5,7 and 1). The corresponding values for milk dessert with 0.1% κ-carrageenan addition were 289 Pa, 244.8 Pa, 218.4 Pa and 226.6 Pa respectively for 0.1%, 0.3%, 0.5% oat gum concentration and control (sample 4, 6, 8 and 2).

The parameters of two examined rheological models Ostwald de Waele and Herschel–Bulkley are collected in Table 2. Herschel–Bulkley model presented the best fit, characterized by highest value of R^2^ (0.933–0.998). It can be seen from both models that all milk desserts exhibited pseudoplastic behavior with flow index less than unity (0.301–0.453 and 0.235–0.58, respectively for Herschel–Bulkley and Ostwald de Waele models). Those n-values obtained in this study are in agreement with values obtained by other research for milk-based desserts with inulin and chickpea flour (Tárrega and Costell 2006; Aguilar-Raymundo and Vélez-Ruiz 2018). Meanwhile, Toker et al. (2013) reported slight lower n-value for milk desserts with different kind of gum. According to Herschel–Bulkley model the milk desserts containing 0.1% κ-carrageenan had the higher structural resistance to flow (higher σ_0_ value observed). The higher σ_0_ value for samples containing 1% κ-carrageenan is indicative of the fact that the forces of the inter-particle link were greater in these samples. Moreover, the κ-carrageenan addition generally caused an increase in K and decreased in n value, when milk desserts with corresponding oat gum concentration are compared. The lower n-values indicating “a more” non-Newtonian nature.

**Table 2:** Rheological parameters (Ostwald de Waele and Herschel–Bulkley models) and thixotropic area for milk dessert systems

Code	Ostwald de Waele model			Herschel–Bulkley model				A_T_ (Pa/s)
	N	K (Pa s^n^)	R^2^	N	K (Pa s^n^)	σ_o_ (Pa)	R^2^	
*Milk desserts without κ*-*carrageenan*								
1-Con	0.580	7.026	0.906	0.330	8.439	16.21	0.985	5677^b^
3-OG01	0.302	33.395	0.958	0.368	29.560	25.09	0.933	9495^a^
5-OG03	0.455	13.090	0.960	0.453	15.567	26.72	0.998	− 2785^d^
7-OG05	0.464	15.669	0.970	0.383	21.36	20.09	0.976	− 2824^c^
*Milk desserts with 0.1% κ*-*carrageenan*								
2-OG0K	0.509	11.595	0.899	0.336	35.148	78.63	0.965	3009^b^
4-G01K	0.235	67.885	0.903	0.301	55.152	125.90	0.955	3113^b^
6-G03K	0.343	31.955	0.901	0.306	42.442	85.05	0.993	3140^b^
8-G05K	0.421	29.925	0.881	0.421	65.232	117.20	0.973	3605^a^

Means in the same column (separate for milk dessert with and without κ-carrageenan) with the same letters aren’t significantly different (Duncan; *p* ≤ 0.05), K (Pa s^n^)—consistency coefficient, n—flow index is dimensionless, σ_o_ (Pa)—yield stress, A_T_—thixotropic area (Pa/s), samples description—as Table 1

According to some research Ostwald–de Waele model can also be fitted well to data obtained for milk desserts with different kind of gums used (Toker et al. 2013; Tárrega and Costell 2006). The K values for this model ranged from 7.026 to 33.395 Pa s^n^ for milk desserts without κ-carrageenan and from 11.595 to 67.835 Pa s^n^ for milk desserts with κ-carrageenan addition. Toker et al. (2013) reported K value in the range from 7.10 to 28.42 Pa s^n^ for milk desserts containing carrageenan, alginate, guar and xanthan gums and their combinations. According to these authors carrageenan had the highest effect on η_50_ (apparent viscosity at shear rate 50 s^−1^), and K value, which is likely to have been resulted from the strong electrostatic interaction between the positively charged region of the milk protein and the negatively charged sulphate groups of carrageenan.

Considering the relative thixotropic area values (A_T_), it can be observed that A_T_ value strongly depend on oat gum concentration especially for milk desserts without κ-carrageenan. However, the effect of oat gum concentration on A_T_ was no linear. A low concentration of oat gum (0.1%, sample 3) caused an increase in the hysteresis loop area, and the sample showed higher A_T_ value than control (sample 1). At a higher oat gum concentration (0.3% and 0.5%; sample 5 and 7, respectively) low loop area can be observed. Similar pattern for milk desserts with 0.1% κ-carrageenan addition was observed. Analyzing differences among desserts samples with the same oat gum concentration, we found that the use of κ-carrageenan stabilizes the effect of oat gum concentration on A_T_ value. Moreover, in this case increase in oat gum concentration increase thixotropic area value (Table 2). Assuming that a thixotropic area value can be used as an index of the energy needed to destroy the structure responsible for flow time dependence, the experimental data indicated that combined addition of oat gum and κ-carrageenan allows to increase the energy needed to breakdown such structure especially at higher oat gum concentration.

### The texture profile analyses (TPA)

TPA analysis has been widely used for rapid evaluation of food texture, however, it should be mentioned that parameters evaluated in TPA are not only material constant but also depend on measurement conditions such as compression speed, temperature and strain (Nishinari et al. 2013). The most important TPA parameter evaluated in the milk desserts is hardness (Szwajgier and Gustaw 2015; Aguilar-Raymundo and Vélez-Ruiz 2018). The textural properties of the milk desserts evaluated in present study changed notably as a function of oat gum and κ-carrageenan addition (Table 3).

**Table 3:** The texture profile analyses test (TPA) of milk dessert

Code	Hardness (N)	Springiness (m)	Cohesiveness (no unit)	Gumminess (N)	Chewiness (J)
*Milk desserts without κ*-*carrageenan*					
1-Con	0.320^f^ ± 0.008	0.942^b^ ± 0.007	0.491^d^ ± 0.013	20.1^d^ ± 0.1	16.7^e^ ± 0.4
3-OG01	0.490^c^ ± 0.011	0.938^b^ ± 0.001	0.589^b^ ± 0.01	25.4^b^ ± 1.2	24.2^b^ ± 0.1
5-OG03	0.417^d^ ± 0.006	0.943^b^ ± 0.003	0.647^a^ ± 0.004	24.06^b^ ± 1.3	23.5^c^ ± 0.3
7-OG05	0.400^e^ ± 0.01	0.953^a^ ± 0.003	0.643^a^ ± 0.003	22.3 ^cd^ ± 0.8	22.3^c^ ± 1.4
*Milk desserts with 0.1% κ*-*carrageenan*					
2-OG0K	0.523^b^ ± 0.014	0.937^b^ ± 0.003	0.529^c^ ± 0.004	21.5 ^cd^ ± 0.38	20.1^d^ ± 0.7
4-OG01K	0.557^a^ ± 0.02	0.937^b^ ± 0.011	0.531^c^ ± 0.003	30.23^a^ ± 2.3	28.38^a^ ± 2.07
6-OG03K	0.526^b^ ± 0.05	0.931^b^ ± 0.011	0.538^b^ ± 0.003	22.13 ^cd^ ± 1.24	20.85 ^cd^ ± 1.46
8-OG05K	0.513^bc^ ± 0.012	0.938^b^ ± 0.001	0.525^c^ ± 0.001	20.67 ^cd^ ± 1.24	20.58 ^cd^ ± 2.06

Values are averages of triplicate determination with standard deviations. Means in the same column with the same letters aren’t significantly different (Duncan; p ≤ 0.05), samples description—as Table 1

The hardness of milk desserts obtained in this study range from 0.32 to 0.49 N for desserts without 0.1% κ-carrageenan addition and from 0.513 to 0.557 N for desserts with κ-carrageenan, which indicate stronger effect of κ-carrageenan on hardness. Combined addition of oat gum and κ-carrageenan allows to obtain milk dessert with stronger texture. However, in both type of tested desserts the highest hardness for desserts with 0.1% oat gum concentration was noted, increased in oat gum concentration above 0.1% decrease hardness. A similar pattern of behavior for gumminess and chewiness was also observed. A high correlation between hardness and gumminess and chewiness for milk dessert was obtained for both type milk desserts tested in the study, which indicates the possibility of limiting the number of parameters tested in TPA (Table 4). The highest hardness, gumminess and chewiness of milk dessert for combined addition of 0.1% oat gum and 0.1% κ-carrageenan were noted, respectively 0.557 N, 30.23 N and 28.38 J. This results indicate high synergistic effect of those two gum at that concentration. Cohesiveness was higher in milk dessert with separate oat gum addition compared to milk dessert with combined addition of oat gum and κ-carrageenan, when desserts with the same oat gum concentration are compared. This result confirm the higher effect of oat gum on viscosity properties compared to κ-carrageenan. As regards springiness, in general, there were no significant differences in springiness, between samples (Duncan test, *p* ≤ 0.05).

**Table 4:** Pearson correlation matrix between rheological parameters of the milk dessert

Variables	H	S	C	G	Ch	A_T_	K	n
*Milk desserts without κ*-*carrageenan*								
H	1.00							
S	− 0.30	1.00						
C	0.57	0.45	1.00					
G	0.97*	− 0.36	0.62	1.00				
Ch	0.92*	− 0.02	0.84	0.94*	1.00			
A_T_	0.46	− 0.75	− 0.46	0.37	0.08	1.00		
K	0.93*	− 0.40	0.27	0.84	0.72	0.73	1.00	
n	− 0.99*	0.34	− 0.47	− 0.94*	− 0.86	− 0.57	− 0.97*	1.00
*Milk desserts with 0.1% κ*-*carrageenan*								
H	1.00							
S	0.03	1.00						
C	0.28	− 0.94*	1.00					
G	0.99*	0.14	0.16	1.00				
Ch	0.95*	0.21	0.07	0.99*	1.00			
A_T_	− 0.51	0.33	− 0.57	− 0.36	− 0.23	1.00		
K	0.86	0.07	0.15	0.91*	0.95*	− 0.02	1.00	
n	− 0.81	0.22	− 0.40	− 0.84	− 0.86	0.08	− 0.96*	1.00

H—hardness, S—springiness, C—cohesiveness, G—gumminess, Ch—chewiness, A_T_—thixotropic area, K—consistency coefficient, n—flow index

*P < 0.1

According to Gustaw et al. (2005) carrageenan affects the increase in hardness of milk desserts but at high concentration (0.3%) may cause adverse changes in milk dessert consistency. The higher hardness of milk desserts obtained with the participation of carrageenan may be the result of the interaction between milk proteins and carrageenan (Mleko and Gustaw 2002). Mleko et al. (1997) suggested that the interaction between these two hydrocolloids took place during heating at temperatures above 60 °C. Other researchers suggest that the improvement of rheological properties of gels with the addition of carrageenan was caused by the separation phase between carrageenan and the proteins (Tárrega et al. 2004; Turgeon and Beaulieu 2001). The hardness of milk dessert noted in presented study was slight higher than that reported by Szwajgier and Gustaw (2015) for dairy dessert with malt, whole and skim milk, who reported a range from 0.18 to 0.33 N. Also Aguilar-Raymundo and Vélez-Ruiz (2018) reported slightly lower hardness for dairy desserts with chickpea flour (0.133–0.391 N).

## Conclusion

A milk dessert with different oat gum concentration and κ-carrageenan addition was prepared and characterized in presented study. Data in this work demonstrated that the mechanical spectra, for all investigated milk desserts were typical for viscoelastic materials such as gels and dispersions (storage module G’ greater than loss module G” values). Our result indicated that the addition of 0.1% κ-carrageenan allows to obtain stronger gel structure compared to milk dessert with separate oat gum addition. Analysis of flow behavior showed the highest shear stress values for milk dessert with 0.1% oat gum concentration, an increase oat gum concentration above that level decreases shear stress, regardless of κ-carrageenan addition. The present study suggested that κ-carrageenan addition can significantly improve viscosity of milk dessert with oat gum, irrespectively to oat gum concentration. Moreover κ-carrageenan addition can stabilized the effect of oat gum concentration on hysteresis loop area; in this case increase in oat gum concentration slightly increase thixotropic area value. Combined addition of 0.1% oat gum and 0.1% κ-carrageenan allows to obtain milk dessert with the highest hardness. Our result showed that 0.1% κ-carrageenan addition can improve rheological properties of milk dessert with oat gum concentration in the range from 0.1 to 0.5%.
